# Synchronization in slowly switching networks of coupled oscillators

**DOI:** 10.1038/srep35979

**Published:** 2016-10-25

**Authors:** Jie Zhou, Yong Zou, Shuguang Guan, Zonghua Liu, S. Boccaletti

**Affiliations:** 1 Department of Physics, East China Normal University, Shanghai 200241, China; 2 CNR-Institute of Complex Systems, Via Madonna del Piano, 10, 50019 Sesto Fiorentino, Florence, Italy; 3 The Embassy of Italy in Tel Aviv, 25 Hamered street, 68125 Tel Aviv, Israel

## Abstract

Networks whose structure of connections evolves in time constitute a big challenge in the study of synchronization, in particular when the time scales for the evolution of the graph topology are comparable with (or even longer than) those pertinent to the units’ dynamics. We here focus on networks with a slow-switching structure, and show that the necessary conditions for synchronization, i.e. the conditions for which synchronization is locally stable, are determined by the time average of the largest Lyapunov exponents of transverse modes of the switching topologies. Comparison between fast- and slow-switching networks allows elucidating that slow-switching processes prompt synchronization in the cases where the Master Stability Function is concave, whereas fast-switching schemes facilitate synchronization for convex curves. Moreover, the condition of slow-switching enables the introduction of a control strategy for inducing synchronization in networks with arbitrary structure and coupling strength, which is of evident relevance for broad applications in real world systems.

Synchronization plays a crucial role in a wide spectrum of technological, biological, and social networks^1^,^2^,^3^,^4^. Even though studied intensively under various aspects^5^,^6^,^7^,^8^,^9^,^10^,^11^, yet a full clarification is needed on the effects of time-dependent coupling structures^12^,^13^,^14^,^15^,^16^, particularly for those circumstances for which the coupling strengths (or connection topologies) may switch in time. For the specific condition (termed as the *blinking network* case) for which the graph structure changes much more rapidly than the units’ dynamics, a comprehensive theoretical framework was established, and applications were developed accordingly^17^,^18^,^19^. However, such an extreme situation does not properly match the generic case, as in the majority of real-world systems the time scale for the coupling evolution turns out to be in fact secular with respect to that of the individual dynamics. For instance, the time scales of synaptic plasticity (the basis of most models in neural circuits) is known to expand from milliseconds to days^20^,^21^,^22^,^23^, and long term plasticity between neurons is actually thought to be the real clue for the higher level functioning of the brain, such as long term memory^24^. A further example is evolutionary processes leading to mutations in genetic networks, which evidently take place over timescales much longer than those related to genetic expressions. Moreover, based on minimal models of interacting binary state nodes, different time scales of coupling switching may even lead the network into distinct phases of collective behavior^25^.

Synchronization in slow-switching networks calls still for a general theory, and for elucidation. Despite the importance of the issue, current studies are so far limited to either simple unit dynamics (e.g. linear system^26^), or simple coupling dynamics (e.g. on-off coupling^27^,^28^), or continuous switching processes^29^. In this paper, we provide instead the general criteria for stable synchronization in slowly varying networked systems, and demonstrate that the stability of synchronization is fully characterized by the largest Lyapunov exponents of the transverse modes associated to the varying structures. Comparison to the fast-switching case allows to show that enhancement of synchronization can be predicted from the profile of the master stability function (MSF). Moreover, a network controlling strategy can be sorted out from our results, which embeds a controlling structure into the system, predesigned as a standard one (i.e. regardless of the specific topology to be controlled), in a way that it may find applications even to systems whose structures are unknown.

## Model and Theory

We start by considering a networked system with *N* identical systems, described as 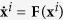, with *i* = 1, 2, 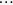, *N*, 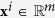, and 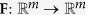. The units interact with their neighbors, so that the dynamics of the *i*-th node is ruled by 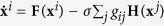, where *σ* is the coupling strength, *g*_*ij*_ is the element of Laplacian **G**, and 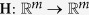 specifies the output function through which coupling is realized among the networks’ units. Let furthermore **x**^1^ = **x**^2^ = … = **x**^*N*^ ≡ **x**^*S*^ be the synchronized solution and *δ***x**^*i*^ ≡ **x**^*i*^ − **x**^*S*^ the *m* dimensional vector accounting for the deviation in time between the evolution of the network’s node *i* and the synchronized state.

For static networks (i.e. when **G** does not change in time), the linearized equation of the system (around **x**^*S*^) is expressed as


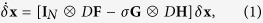


where *δ***x** = (*δ***x**^1^, 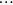, *δ***x**^*N*^)^T^, and *D***F** and *D***H** are the Jacobian functions of **F** and **H**, respectively, evaluated on the synchronized solution **x**^*S*^. By plugging 
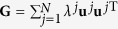
 into Eq. (1) (where *λ*^1^ = 0 < *λ*^2^ < 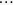 < *λ*^*N*^ are the eigenvalues and {**u**^*j*^} the corresponding eigenvectors providing an orthonormal basis of 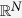), and setting *δ***x**(*t*) = **Q***δ***y**(*t*) [with **Q** = (**u**^1^, 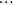, **u**^*N*^)⊗**I**_*m*_) and *δ**y*** = (*δ**y***^1^, 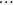, *δ**y***^*N*^)^T^], one obtains


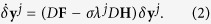


The largest Lyapunov exponent of the transverse mode *δ***y**^*j*^ is defined as


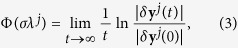


which is a function of the eigenvalue *λ*^*j*^ and coupling strength *σ*, also known as the Master Stability Function (MSF). The necessary condition for having a stable synchronization is that MSF Φ(*σλ*^*j*^) is negative for all the transverse modes *j* ≥ 2, as elaborated in ref. 11.

Now, let us consider the case of time-varying networks where the Laplacian **G** is composed of a sequence of coupling matrices (**G**_1_, **G**_2_, 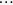) that occur in a chronological order. Suppose, further, that **G**_*k*_ lasts for a lapse *τ*_*k*_ under coupling strength *σ*_*k*_ (a condition that we will denote as {**G**_*k*_, *τ*_*k*_, *σ*_*k*_}). Then **G**(*t*) is represented by **G**(*t*) = ({**G**_1_, *τ*_1_, *σ*_1_}, {**G**_2_, *τ*_2_, *σ*_2_}, 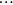).

To elucidate the effect of a slow switching, we start from the simplest case that the network alternates between two structures (**G**_1_ and **G**_2_), so that the system periodically switches between configurations {**G**_1_, *τ*_1_, *σ*_1_} and {**G**_2_, *τ*_2_, *σ*_2_}, with a period *T* = *τ*_1_ + *τ*_2_. Again taking 
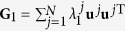
 and 
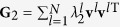
, one obtains that the necessary condition of stable synchronization is the following: for each pair of transverse modes (*j, l* ≥ 2), if *S*_*lj*_ ≠ 0 where *S*_*lj*_ = **v**^*l*T^**u**^*j*^, one should have (see Methods, part I for details)


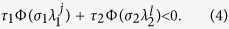


The condition is readily extended to the general case of a network that slowly switches among a sequence of *L* configurations, i.e. ({**G**_1_, *τ*_1_, *σ*_1_}, 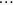, {**G**_*L*_, *τ*_*L*_, *σ*_*L*_}) with 
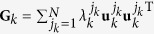
: for each combination of the transverse modes of the *L* configurations (*j*_1_, *j*_2_, 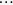, *j*_*L*_), if all the *L* − 1 pairs of vectors (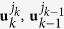) (2 ≤ *k* ≤ *L*) are non-orthogonal, one should have


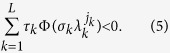


With the conditions (4, 5), for any function 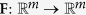 the MSF Φ(*σλ*^*j*^) is, indeed, obtained straightforwardly, and once one has the specific sets of eigenvalues and eigenvectors that diagonalize the *L* configurations, the specific values of 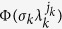 can be simply read on the *unique* MSF for each 2 ≤ *k* ≤ *L*, so that predictions on the stability of synchronization can be elegantly made by checking whether conditions (4, 5) are fulfilled. In the following, we will therefore linger on some practical applications of our theory.

## Results

In this paper, we consider the case of Rössler oscillators where the unit dynamics is described by 
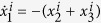
, 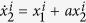, 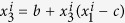 with 
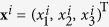
 and parameters being *a* = *b* = 0.2, and *c* = 7. **H** is a 3 × 3 matrix, whose elements are 1 for *h*_11_ and 0 otherwise, i.e it realizes a linear coupling on the *x*_1_ variable of the graph’s units. Dynamics is integrated with a fixed time step Δ*t* = 0.001, and the synchronization performance is evaluated by the error function defined as


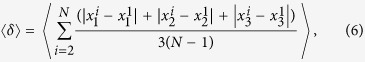


where 〈⋅〉 denotes the time average over a sufficient long time after discarding transient processes.

### Validation of Analytic Predictions

**Figure 1:**
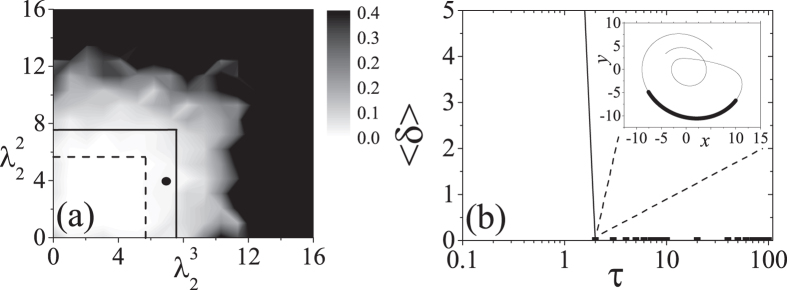
(**a**) Gray plot of 〈*δ*〉 in the (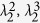) plane, for 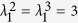 and *σ*_1_ = *σ*_2_ = 1. Solid lines indicate the predicted boundaries of the synchronization region. Dashed lines indicate the boundaries for the case of fast switching. 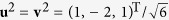, and 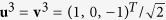. (**b**) 〈*δ*〉 *vs. τ* for the case 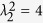 and 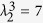 [indicated by a solid dot in panel (a)]. Inset: Thick black curve shows a typical trajectory of the Rössler oscillator projected on *xy*-plane for the lapse of time *τ* = 2. Thin line reports the actual continuation of that trajectory, and serves as a guide for eye.

To validate the analytic prediction, we first consider a simple case of a network made of just three oscillators, which switches between two structures along the same time interval *τ*. The Laplacians of the two connection arrangements are 
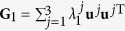
 and 
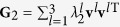
 with **u**^1^ = **v**^1^ = (1, 1, 1)^T^ and 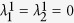 (which is along the synchronization manifold **x**^1^ = **x**^2^ = **x**^3^). For simplicity, we set *σ*_1_ = *σ*_2_ = 1, **u**^2^ = **v**^2^, and **u**^3^ = **v**^3^, and thus *S*_*jl*_ = *δ*_*jl*_. According to Eq. (4), the system is stable on the synchronization manifold when 
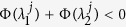
 for *j* = 2 and 3, respectively. Figure 1(a) reports the synchronization index 〈*δ*〉 in the (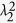, 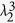) plane, where the values of 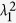 and 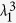 are fixed as 3. Notice that the solid lines [obtained from the equations 
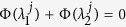
 for *j* = 2 and 3, respectively] separate the plane into two regions: the white one (synchronized dynamics) and the dark one (asynchronous behavior), in good agreement with the numerical results. It is remarkable that this result is pertinent to a specific choice for the switching time (*τ* = 10). Since this result pertains to the slow-switching scenario, it is worth exploring the range of *τ* for which the overall scenario is actually maintained. Figure 1(b) reports 〈*δ*〉 *vs. τ*, for 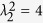 and 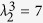 [the solid spot in (a)]. 〈*δ*〉 vanishes for *τ* ≥ 2, whereas it diverges for *τ* < 2 (i.e. when the structures start to switch faster).

In fact, as soon as the switching process occurs over fast enough time scales, a sufficient condition is that the time average of the coupling matrices 
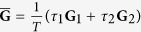
 supports synchronization^17^,^18^. In particular, when **G**_1_ and **G**_2_ share the same basis, i.e. **u**^*j*^ = **v**^*j*^ for all *j*, an equivalent condition for fast-switching networks can be obtained, that is: 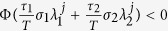 for 2 ≤ *j* ≤ *N* (see Methods, part II for details). For comparison, this latter condition is reported with dashed lines in Fig. 1(a). As the region delimited by the dashed lines is embraced in that contoured by solid lines, we may conclude that in this case a slow switching scheme is prompting synchronization more than a fast switching one.

### The Shape of Master Stability Function

A second remarkable application is the prediction, under fixed topology and switching coupling strengths, of the most favorable switching scheme for synchronization. The conditions of slow (large *τ*) and fast (small *τ*) switching schemes lead, indeed, to a general criterion for a class of networks whose coupling strength may vary with time while the Laplacian **G** is fixed, for example networks with on-off coupling^27^. Specifically, for the case that the coupling strength switches between two values *σ*_1_ and *σ*_2_ alternatively, the criterion (which can be readily extended to general case of arbitrary number of different values) can be stated as follows: when the MSF is *concave* in the domain spanned by the Laplacian eigenvalues, i.e. *α*Φ(*x*_1_) + (1 − *α*)Φ(*x*_2_) ≤ Φ[*αx*_1_ + (1 − *α)x*_2_], if the network supports a synchronous dynamics under a fast switching [i.e. 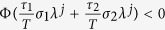 ( ∀ *j* ≥ 2)], the same network will also behave synchronously under slow switching of the same structures [i.e. 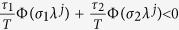 ( ∀ *j* ≥ 2)]. When instead the MSF is convex, the situation is reversed, and slow switching conditions include the fast switching ones.

In order to better elucidate the above relationship between the two switching schemes, we take the case of a complete network, for which all non-vanishing eigenvalues of the Laplacian are the same, *λ*^2^ = *λ*^3^ = 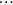 = *λ*^*N*^ = *N*, so that the *N* − 1 transverse eigenmodes form an (*N* − 1)-dimensional linear subspace. Specifically, we preserve the network structure as a complete graph, and switch the coupling strength between *σ*_1_ and *σ*_2_ with the same time interval *τ*, so that the argument *σλ* of the MSF is (for all transverse modes) switched between *σ*_1_*N* and *σ*_2_*N*. Noting that the associated conditions for stable synchronization are 
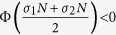
 (fast switching) and 
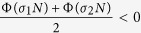
 (slow switching).

**Figure 2:**
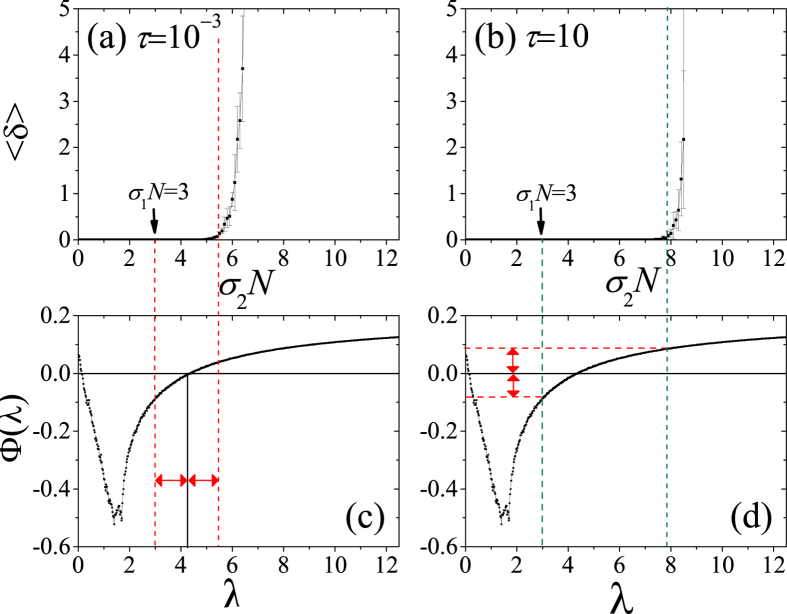
Upper panels: 〈*δ*〉 *vs. σ*_2_*N* for *τ* = 10^−3^ [fast switching, panel (a)] and *τ* = 10 [slow switching, panel (b)]. *N* = 100 and *σ*_1_*N* = 3. Lower panels: the MSF corresponding to our choice of the Rössler chaotic oscillator. In panel (c), the red dashed lines pointed by the red arrows indicate the value of *σ*_1_*N* = 3 (the left one), and *σ*_2_*N* which satisfies 
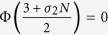
 (the right one), therefore giving the equal length of the two arrows. In panel (d), the red dashed lines pointed by the red arrows indicate the value of Φ(*σ*_1_*N* = 3) (the lower one), and Φ(*σ*_2_*N*) which satisfies 
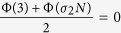
 (the upper one), and also the equal length of the two arrows. The green dashed lines indicate the corresponding values of *σ*_1_*N* = 3 and *σ*_2_*N*. The results are averaged over 50 realizations.

Figure 2 reports the results for the cases of *τ* = Δ*t* = 0.001 (fast switching, panel (a)) and *τ* = 10^4^Δ*t* = 10 (slow switching, panel (b)), where *σ*_1_ is fixed at 3/*N* so that *σ*_1_*N* = 3, and *σ*_2_*N* goes from 3 to 15 (being the MSF concave in the corresponding domain). The MSF for the considered Rössler chaotic dynamics is shown in panels (c) and (d). The long dashed lines crossing panels (a) (red lines) and (b) (green lines) show the boundaries of *σ*_2_*N* for synchronization, coming from the conditions 
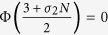
 and 
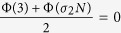
, respectively. Dashed lines in (c) and (d) indicate how one can predict the boundaries with just the help of the shape of the MSF (details in the caption). Theory and the numerical results match very well. It is clearly shown that the range spanned by the green dashed lines in panel (d) is larger than that spanned by the red dashed line in panel (c), which confirms the our conclusion that when the MSF is concave in the domain concerned, the case of slow switching allows synchronization for a range of *σ*_2_*N* larger than that of fast switching. While this example considers explicitly a concave MSF, the case of a convex MSF is described and reported in the Supplementary Information.

### A Control Strategy

As a last step of our study, we show how the discovered conditions suggest the realization of controlling strategies for inducing synchronization in generic networks. Specifically, we introduce a heuristic approach, whose main idea is to periodically add a controlling network into a pristine graph, in a way that the whole network switches alternatively between the original topology (i.e. the target network to be controlled) and the controlling structure. This latter structure is actually chosen in order that the resulting time-evolving graph satisfies the conditions for stable synchronization provided in the above. In fact, the spectrum of the Laplacian of the original network may be very different from one to another, with more heterogeneous graphs displaying larger ranges of eigenvalues’ spectrum^30^. Thus, finding a proper controlling structure for each specific original network is a key step in this strategy.

Having to cope with a generic case, we here propose a controlling structure (a graph) for which the non null eigenvalues of the Laplacian are all the same, say *λ*, and the coupling strength *σ* is chosen such that Φ(*σλ*) = Φ_min_ (where Φ_min_ is the minimum value of the MSF). Because the values of MSF for all the transverse modes reach Φ_min_, this kind of networks is optimal for synchronization, and we refer to them as *Optimal synchronization networks* (OSN). For binary interaction networks (those represented by adjacency matrices), the number of optional OSNs could be very large. A practical way of constructing an OSN is provided in ref. 31.

Now suppose that, during each period, the pristine network stays fixed for a time *τ*_U_, and then the system switches to an OSN which remains fixed for a time *τ*_O_. Noted that, regardless the specific spectrum of the original graph, the possible values of Φ will never exceed the maximum value of the MSF, Φ_max_. Therefore, according to our condition for slow switching, the graph will be controlled when *τ*_O_/*τ*_U_ ≥ Φ_max_/|Φ_min_|, no matter what its structure is. For our specific choice of Rössler system, 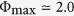 and 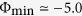, one may expect that *any* network can be controlled when 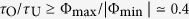.

**Figure 3:**
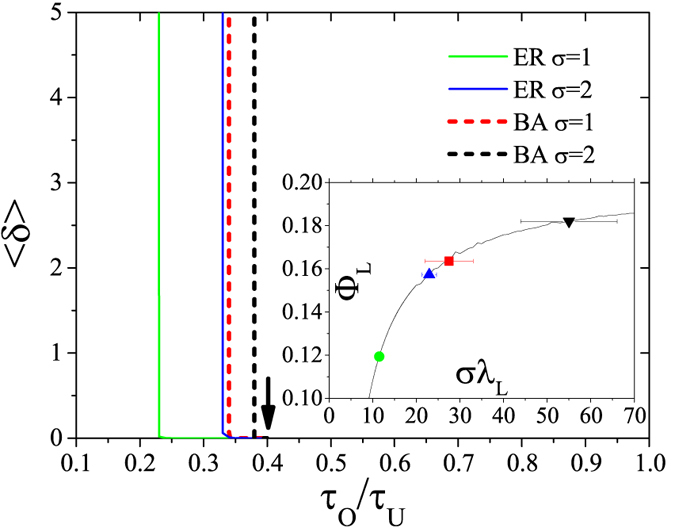
〈*δ*〉 as a function of the ratio *τ*_O_/*τ*_U_ with *τ*_O_ fixed at *τ*_O_ = 10, for ER and BA networks with different coupling strength *σ* (see the legend for the color code). The arrow indicates the value of 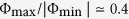. The inset shows the largest MSF value Φ_L_ of these networks. The reported results refer to ensemble averages over 50 different realizations.

To validate our predictions, we generate homogeneous and heterogenous networks of size *N* = 100 by means of the Erdös-Rényi (ER^32^) and Barabási-Albert (BA^33^) models, and adopt complete networks as controlling structures (other choices of OSNs were considered and similar results, not shown here, were obtained). According to the condition for slow switching, the threshold of the ratio *τ*_O_/*τ*_U_ for the controlling procedure is fully determined by the transverse mode which has the largest MSF value among all the others. By denoting the largest MSF value reached by this mode as Φ_L_, the synchronization condition requires *τ*_O_/*τ*_U_ ≥ Φ_L_/|Φ_min_|. The inset of Fig. 3 reports the values of Φ_L_. When *σ* = 1, Φ_L_ is relatively small for both ER and BA networks, and therefore the graphs can be controlled with a small ratio *τ*_O_/*τ*_U_(≥Φ_L_/|Φ_min_|), as shown in the main panel of Fig. 3. When *σ* = 2, the value of Φ_L_ increases, and a larger ratio of *τ*_O_/*τ*_U_ is needed. However, a further increase in *σ* leads to the saturation of Φ_L_ at Φ_max_, and to the convergence of the ratio *τ*_O_/*τ*_U_ to the expected threshold value (about 0.4), as indicated by the arrow in the main panel of the figure.

## Discussion

In conclusion, we succeeded in setting the necessary condition for stable synchronization of slowly switching networks. Together with the criterion of fast switching networks in previous works, the both ends of the related condition of switching networks now have been filled. As a contrast to fast switching networks, for slowly switching networks, the condition of stable synchronization is affected by the largest Lyapunov exponent of each transversal mode of switching structures rather than that of their aggregation. Interestingly, we find that such difference in the two switching schemes help us, with the shape of the Master stability function (MSF), in predicting which switching scheme is more favourable for synchronization. Specifically, when the MSF is concave (convex) in the domain the argument span, the network supports a synchronous dynamic under a fast (slow) switching, the same network also behaves synchronously under slow (fast) switching of the same structures. This result further exploits the information embedded in the MSF, from the number and positions of the null points to the shape of it.

The results about slow switching networks may provide potential applications. In this paper, we propose a heuristic controlling strategy according to the characteristic of slow switching networks, where a controlling structure is inserted to the target network periodically. To make this strategy to be general for different situations, we introduce the optimal synchronization networks (OSN) (graphs whose largest Lyapunov exponents of all the transverse modes are close to the minimum value of the Master Stability Function) and show that they serve as an excellent class of controlling networks. In particular, with the knowledge of MSF, the ratio between the action times of OSN and target networks could be preassigned as a standard, and the target networks could be controlled regardless of their specific structure or coupling strength. Therefore, this strategy has potential applications to a wide variety of systems, with unbounded coupling and unknown structure.

We highlight that the obtained condition of stable synchronization is *necessary* but not *sufficient*, which roots from the fundamental limitation of MSF which only assesses local stability of the synchronized state. Sufficient conditions may, in limited situations, be derived from global stability analysis, such as Lyapunov function methods.

## Methods

### Condition of stable synchronization of slowly switching networks

Let us start from the original case of a network which is fixed on a single structure. While the condition, for this case, has been obtained by Pecora *et al*. in ref. 11, we shall further elaborate on the details of the analytic procedure, which will be helpful to further understand the general case of slow-switching networks.

The linearized equation of the system deviating around the solution **x**^*S*^ ≡ **x**^1^ = **x**^2^ = .... = **x**^*N*^ is expressed as


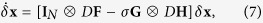


where the meaning of the notations are the same as those in the main text. The Laplacian **G** could be decomposed as 
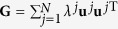
 where {*λ*^*j*^} is the spectrum with *λ*_1_ = 0 and {**u**^*j*^} the eigenvectors with **u**^1^ corresponding to the synchronization manifold. Taking now **I**_*N*_ as 
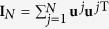
, one gets


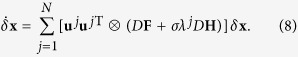


Setting *δ***x**(*t*) = **Q***δ***y**(*t*) with **Q** = (**u**^1^, 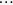, **u**^*N*^)⊗**I**_*m*_, Eq. (8) becomes


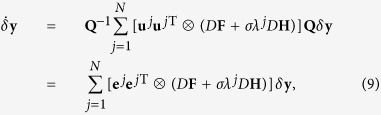


where **e**^*j*^ is a unit vector whose *j*-th element is 1 and 0 otherwise. The matrix in Eq. (9) is block diagonal with *m* × *m* blocks. Taking *δ***y** as *δ***y** = (*δ***y**^1^, 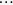, *δ***y**^*N*^) with 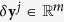, one has


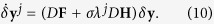


Generally, *D***F** + *σλ*^*j*^*D***H** varies with the evolution of the state **y**^*j*^, so we can denote *D***F** + *σλ*^*j*^*D***H** as 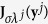. Since 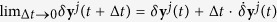, the solution of *δ***y**^*j*^ can be expressed as


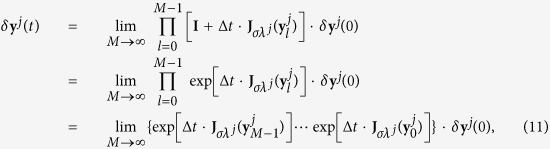


where *δ***y**^*j*^(0) is the initial condition, Δ*t* = *t*/*M*, and 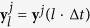. The *M* → ∞ limit procedure gives the formal integral


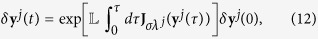


where 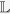 stands for time-ordered integration, reflecting the continuum limit of the successive left multiplications in Eq. (11). Denoting 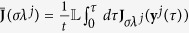, Eq. (12) becomes


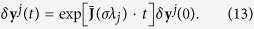


Now, for ergodic systems, 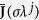 is asymptotically (i.e. at large enough *t*) independent of *t*, and


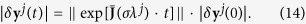


Therefore, if one defines as the largest Lyapunov exponent associated to the *j*-th eigen-mode


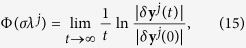


one has 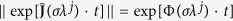 when *t* is large. Stable synchronization requires (as a necessary condition) the largest Lyapunov exponents Φ(*σλ*^*j*^) to be negative for all the transverse eigen-modes (*j* ≥ 2).

Finally, according to Eq. (13), the solution of *δ***y** is


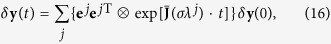


and therefore


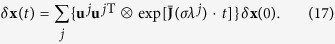


Suppose now that a network periodically switches between two configurations {**G**_1_, *τ*_1_, *σ*_1_} and {**G**_2_, *τ*_2_, *σ*_2_}, with *τ*_1_ and *τ*_2_ being the lasting times of the two configurations and *σ*_1_ and *σ*_2_ the coupling strengths, respectively. Similarly to the above, one can decompose **G**_1_ and **G**_2_ as 
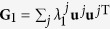
 and 
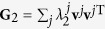
. For each period *T* = *τ*_1_ + *τ*_2_ (supposing **G**_1_ acts first), the state *δ***x** attained by at time *T* can be written as


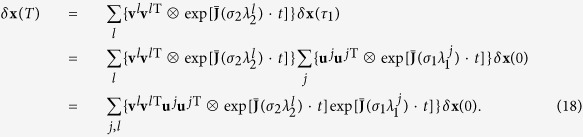


We define *S*_*lj*_ = **v**^*l*T^**u**^*j*^, so that **v**^*l*^**v**^*l*T^**u**^*j*^**u**^*j*T^ = ∑_*j*′_**u**^*j*′^**u**^*j*T^*S*_*j*′*l*_*S*_*lj*_. Then Eq. (18) can be rewritten as


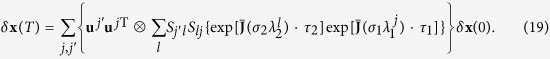


Taking again *δ***x** = **Q***δ***y**, one eventually gets


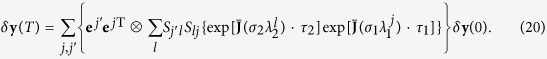


Remarkably, when **e**^*j*′^**e**^*j*T^ acts on a vector, it actually picks its component on the direction **e**^*j*^ and turns it into the direction **e**^*j*′^. Thus, one obtains


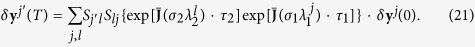


When *τ*_1_ and *τ*_2_ are large, we have


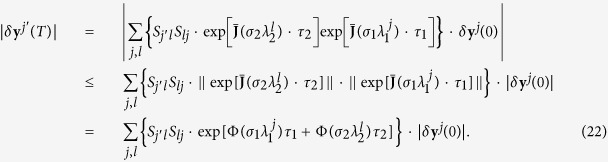


The stable synchronization demands the convergence of |*δ***y**^*j*′^(*T*)| which accounts for the condition that: for each pair of (*j, l* ≥ 2), if *S*_*lj*_ ≠ 0, then one should have


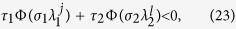


as stated in Eq. (4). In analogy, the condition could be directly extended to the general case of arbitrary number of configurations, as formulated by Eq. (5).

### Condition of stable synchronization of fast switching networks

For the case of fast-switching, we also start from the situation that a network periodically switches between two configurations {**G**_1_, *τ*_1_, *σ*_1_} and {**G**_2_, *τ*_2_, *σ*_2_}, while here *τ*_1_ and *τ*_2_ are sufficiently small. It has been found that the condition of stable synchronization for fast-switching networks is that the time average of the coupling matrices 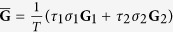 supports synchronization^17^.

Now, we look at a specific case that 
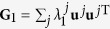
 and 
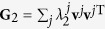
 share the same basis, i.e. **u**^*j*^ = **v**^*j*^ for all *j* and therefore *S*_*lj*_ = *δ*_*lj*_. In this case, stable synchronization could be attained when for all the transverse modes one has


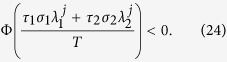


This result is readily extended to a general situation of networks fast switching among arbitrarily number of configurations. The condition of stable synchronization now is


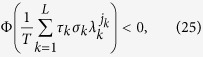


where notations are similar to those in the Eq. (5) of the main text. We note that this case that the Laplacians of all the configurations share the same basis covers a large class of network whose coupling strength varies with time but structure fixes.

## Additional Information

**How to cite this article**: Zhou, J. *et al*. Synchronization in slowly switching networks of coupled oscillators. *Sci. Rep.* **6**, 35979; doi: 10.1038/srep35979 (2016).

## Supplementary Material

Supplementary Information
