# Supplementary material for: Synchronization in slowly switching networks of coupled oscillators
Source: Sci Rep. 2016 Oct 25;6:35979. doi: 10.1038/srep35979 (PMC5078792; doi:10.1038/srep35979)
Supplement: Supplementary Information [file srep35979-s1.pdf]

Supplementary Information:
Synchronization in slowly switching networks of coupled oscillators

Jie Zhou,[1] Yong Zou,[1] Shuguang Guan,[1] Zonghua Liu,[1] and S. Boccaletti[2,3]

[1]*Department of Physics, East China Normal University, Shanghai 200241, China*
[2]*CNR-Institute of Complex Systems, Via Madonna del Piano, 10, 50019 Sesto Fiorentino, Florence, Italy*
[3]*The Embassy of Italy in Tel Aviv, 25 Hamered street, 68125 Tel Aviv, Israel*

(Dated: September 19, 2016)

THE CASE OF CONVEX MASTER STABILITY FUNCTION

As indicated in the main text, when the Master stability function (MSF) in the domain spanned by the Laplacian eigenvalues is *convex*, i.e. $\Phi[\alpha x_1 + (1-\alpha)x_2] \leq \alpha\Phi(x_1) + (1-\alpha)\Phi(x_2)$, if the network supports a synchronous dynamics under a slow switching [i.e. $\frac{\tau_1}{T}\Phi(\sigma_1\lambda^j) + \frac{\tau_2}{T}\Phi(\sigma_2\lambda^j) < 0$ $(\forall j \geq 2)$], the same network will also behave synchronously under fast switching of the same structures [i.e. $\Phi(\frac{\tau_1}{T}\sigma_1\lambda^j + \frac{\tau_2}{T}\sigma_2\lambda^j) < 0$ $(\forall j \geq 2)$]. Here, we provide an example of a convex MSF, by adopting the Chua's circuit system, whose oscillatory dynamics is

$$\begin{cases} \dot{x} = \alpha[y - x + f(x)] \\ \dot{y} = x - y + z \\ \dot{z} = -\beta y - \gamma z \end{cases} \tag{1}$$

with $\alpha = 10$, $\beta = 14.87$, $\gamma = 0$, and

$$f(x) = \begin{cases} \dot{x} = -bx - a + b, & x > 1 \\ \dot{y} = -ax, & |x| < 1 \\ \dot{z} = -bx + a - b, & x < -1 \end{cases} \tag{2}$$

where $a = -1.27$ and $b = -0.68$ [1].

The MSF of the Chua's circuit system is shown in Fig. 1 (c) and (d), where the output function is still taken as $h_{11} = 1$ and 0 otherwise. One easily sees that the shape of the MSF is indeed convex. Similarly to what reported in the main text, we consider here a complete network of size N where the coupling strength switches between σ_1 and σ_2 with the same time interval τ. Figure 1 (a) and (b) show the synchronization performance for the cases of fast switching ($\tau = 0.001$) and slow switching ($\tau = 10$), respectively, where σ_2 is fixed as $\sigma_2 N = 1$ and $\sigma_1 N$ changes from 0 to 30. With the help of dashed lines, one realizes that the feasible range of σ_1 for stable synchronization matches well our theoretical predictions, i.e. $\Phi(\frac{\sigma_1 N + \sigma_2 N}{2}) < 0$ for fast switching and $\frac{\Phi(\sigma_1 N) + \Phi(\sigma_2 N)}{2} < 0$ for slow switching. In this example it is therefore evident that the feasible range of σ_1 for the case of fast switching includes that corresponding to the case of slow switching, as theoretically predicted.

[1] T. Matsumoto, L. O. Chua, and M. Komuro, The double scroll, *IEEE Trans. Circuits Syst.* **32**, 797 (1985).

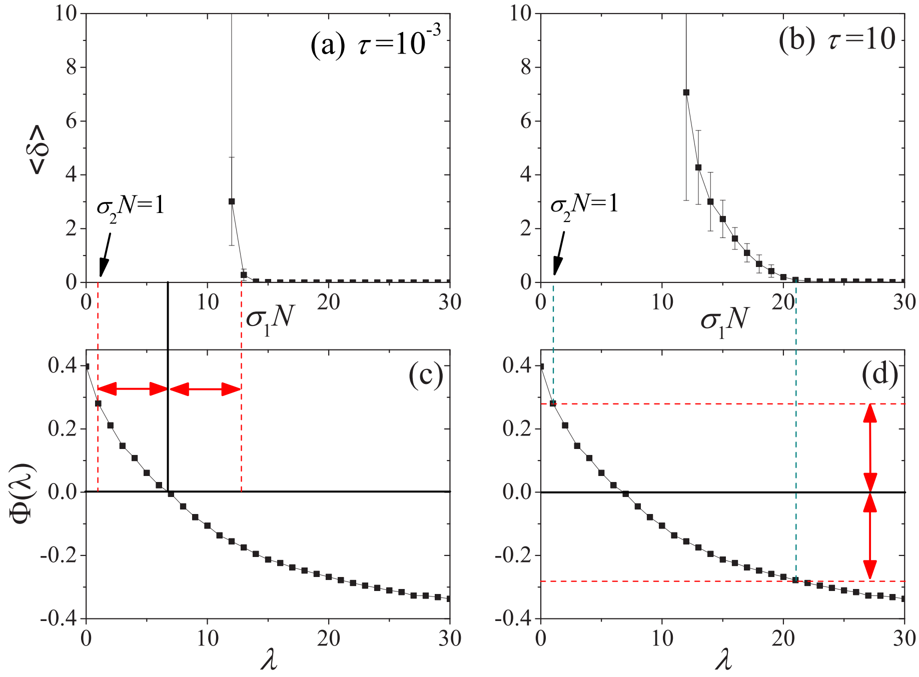

FIG. 1: (Color online). Upper panels: $\langle\delta\rangle$ *vs.* $\sigma_1 N$ for $\tau = 10^{-3}$ [fast switching, panel (a)] and $\tau = 10$ [slow switching, panel (b)]. $N = 100$ and $\sigma_2 N = 1$. Lower panels: the MSF corresponding to our choice of the Chua's circuit oscillator. In panel (c), the red dashed lines pointed by the red arrows indicate the value of $\sigma_2 N = 1$ (the left one), and $\sigma_1 N$ which satisfies $\Phi(\frac{1+\sigma_1 N}{2}) = 0$ (the right one), therefore giving the equal length of the two arrows. In panel (d), the red dashed lines pointed by the red arrows indicate the value of $\Phi(\sigma_2 N = 1)$ (the upper one), and $\Phi(\sigma_1 N)$ which satisfies $\frac{\Phi(1)+\Phi(\sigma_1 N)}{2} = 0$ (the lower one), and also the equal length of the two arrows. The green dashed lines indicate the corresponding values of $\sigma_2 N = 1$ and $\sigma_1 N$. The results are averaged over 50 independent realizations.
